# Dementia Care Involvement and Training Needs of Social Services Directors in U.S. Nursing Homes

**DOI:** 10.1093/geroni/igab046.445

**Published:** 2021-12-17

**Authors:** Jung Kwak, Kevin Smith, Mercedes Bern-Klug

**Affiliations:** 1 The University of Texas at Austin, Austin, Texas, United States; 2 University of Iowa, Iowa City, Iowa, United States

## Abstract

This study describes social services directors’ involvement in dementia care in U.S. nursing homes, focusing on interest in and needs for dementia care training. Respondents were 841 social service directors from U.S. nursing homes. We found that 87% of social service departments engaged in cognitive assessment; 59% of social services directors were strongly interested in dementia care training, and 23% would need up to 10 hours of preparation time or would not be able to train staff on dementia-related care. Racial minority background, fewer years of experience in nursing homes, and barriers to staffing predicted strong interest in dementia care training. These findings demonstrate social services directors’ active involvement in dementia care and need for training.

